# Weight reduction and dietary improvements in a cluster-randomised controlled trial for adults with intellectual disabilities

**DOI:** 10.29219/fnr.v67.9505

**Published:** 2023-12-19

**Authors:** Helen K. Røstad-Tollefsen, Svein O. Kolset, Kjetil Retterstøl, Heidi Hesselberg, Marianne Nordstrøm

**Affiliations:** 1 Department of Nutrition, Institute of Basic Medical Sciences, University of Oslo, Oslo, Norway; 2 Baerum Municipality, Housing, Activity and Organized Work, Health, and Welfare, Baerum, Norway; 3 Lipid Clinic, Oslo University Hospital, Oslo, Norway; 4 Frambu Resource Centre for Rare Disorders, Siggerud, Norway; 5 Unit for Inborn and Hereditary Neuromuscular Disorders, Department of Neurology, Oslo University Hospital, Oslo, Norway

**Keywords:** intellectual disability, behaviour change techniques, weight reduction, dietary habits, nudging, fruit, vegetables

## Abstract

**Background:**

People with intellectual disabilities (IDs) have an increased risk of obesity and health concerns related to their nutritional status and dietary intake.

**Objective:**

To assess the effectiveness of a multi-component intervention on weight, waist circumference (WC), clinical health parameters and dietary habits in a group of overweight and obese adults with mild-to-moderate ID.

**Design:**

A 7-month cluster-randomised trial and a 7-month follow-up of the intervention group after the end of intervention when the group received usual care. The intervention consisted of monthly dietary-group courses tailored to the participants’ cognitive abilities and practical skills, monthly nutritional courses for staff, use of behaviour change techniques and nudging. The control group received usual care during the intervention.

**Results:**

There were 32 participants aged 22–61 years: 15 in the intervention group and 17 in the control group. After 7 months, a non-significant weight difference (median difference = −1.25 kg; 95% confidence interval [CI] = −2.00; 0.95 vs. +1.00 kg; CI = −1.15; 3.00, *P* = 0.08) and a significant WC difference were observed between the intervention and control groups (median difference = −3.75 cm; CI: −7.68; 0.11 vs. 0 cm; CI = −3.99; 1.00, *P* = 0.03), respectively. The median reduction in WC continued in the intervention group during the 7-month follow-up (median difference = −7.50 cm; CI: −13.57; −3.16, *P* = 0.002). A significant difference in frequency intake of fruit (*P* = 0.03) and berries (*P* = 0.004) was observed between the groups after 7 months, supported by a significant increase in measured serum-carotenoid levels in the intervention group after 7 months (median difference = 0.26 mmol/L; CI: −0.12; 0.52, *P* = 0.007).

**Conclusions:**

A significant difference in WC was observed between the groups, accompanied by changes in blood parameters and dietary habits.

## Popular scientific summary

- A multi-component cluster-randomised controlled study tailored to persons with mild-to-moderate intellectual disabilities, targeting them and their support staff.
- Difference in waist circumference was observed between the groups after 7 months, continuing to decrease in the intervention group at follow-up.
- The frequency intake of fruit and berries differed between the groups after 7 months, in line with an increase of serum-carotenoid levels in the intervention group after 7 months and the follow-up period.

People with intellectual disabilities (IDs) experience a higher disease burden than the general population, resulting in several health issues related to nutritional status and dietary intake (1–3). Notably, the prevalence of obesity in this group is approximately three times higher than the general population (3, 4), especially amongst individuals with mild-to-moderate ID living in municipal care homes and some genetic syndromes such as Down syndrome (5–7). Moreover, these individuals face an elevated risk of poor dietary habits and nutritional deficiencies (8, 9), and studies have consistently indicated a high consumption of sweets, snacks and soft drinks and a low intake of fruits and vegetables in this group (10, 11).

Few multi-component weight intervention studies have been tailored to this group (12). However, some studies have shown promising results in improving work routines and increasing physical activity (13, 14) but only modest effects on promoting healthy eating patterns and reducing weight and waist circumference (WC) in overweight persons with IDs (15, 16). Therefore, it is recommended to implement multi-component health interventions tailored to the cognitive, communicative and health literacy abilities of this population. These interventions should incorporate behaviour change techniques (BCTs) and provide training to both residents and their staff to address these challenging issues (14–18).

Staff members working with individuals with ID in municipal care homes are uniquely positioned to positively influence health behaviours and dietary choices. Nevertheless, studies have underscored their need for enhanced nutritional knowledge (18, 19). To improve services and promote healthy diets in residents, staff members also need systems to establish common ground on healthy foods, develop skills to facilitate healthy diets, acquire practical cooking skills and apply dietary knowledge (20).

Based on the challenging background of high prevalence of obesity, poor dietary habits, low nutritional knowledge and a need for evidence-based measures to address these issues, we conducted a small explorative multi-component randomised controlled trial (RCT). The primary aim was to assess the effectiveness of this intervention over a 7-month period on weight in a group of overweight and obese adults with mild-to-moderate ID living in municipal care homes, compared to a control group receiving usual care. The secondary aim was to assess the effectiveness of the intervention on WC, clinical health parameters and dietary habits in the intervention group compared to the control group after 7 months. Finally, we aimed to study the long-term effectiveness of the intervention in the intervention group after a 7-month follow-up after the end of the intervention, when the intervention group had received usual care.

## Present investigation

### Study design

The study was a 7-month cluster-RCT conducted in a municipality in south-eastern Norway. After 7 months, the intervention group was asked to complete a 7-month follow-up period with no active intervention. The control group was asked to continue their normal routines during the intervention. In the follow-up period, the control group was offered the same dietary course as the intervention group had undertaken, but no other components of the intervention. Therefore, the comparison of the groups after 14 months is less relevant. Offering the control group, the dietary courses were done in response to a request from the Committees for Medical and Health Research Ethics (REK) to provide some benefits to the control group for participating in this study. More resources than available would have been needed to perform a crossover study.

We started the intervention in March 2020 at the beginning of the COVID-19 pandemic; however, we had to pause and restart the intervention in October 2020. The study ended in November 2021. The inclusion criteria were individuals with mild-to-moderate ID defined according to the Tenth Revision of the International Statistical Classification of Diseases and Related Health Problems (ICD-10) (21). Participants needed to be 18 years or older and live in or in connection with a municipal care home in the municipality where this study was conducted and have a body mass index (BMI) ≥ 27 kg/m^2^. Information on the ID levels of the participants was retrieved from their medical journals in their municipal care homes. The exclusion criteria were the need for a special diet due to an underlying disease or condition (e.g. a ketogenic diet for treating epilepsy or Prader-Willis syndrome). Furthermore, patients with insulin-treated diabetes, malignant disease, known kidney failure or blood pressure (BP) > 180/100 mmHg were excluded.

### Recruitment and participants

Information about the study was disseminated by managers in municipality-based health and care services for individuals with ID. An easy-to-read informed consent form and a video were produced and distributed through internal websites to inform all potential participants. Additionally, an information letter was developed for the guardians of the participants. The staff working with the participants checked their legal rights in their medical journals at the municipal care homes and determined whether they could consent independently. A written informed consent was obtained from all participants and, if relevant, together with their legal guardians.

### Randomisation

**Fig. 1:**
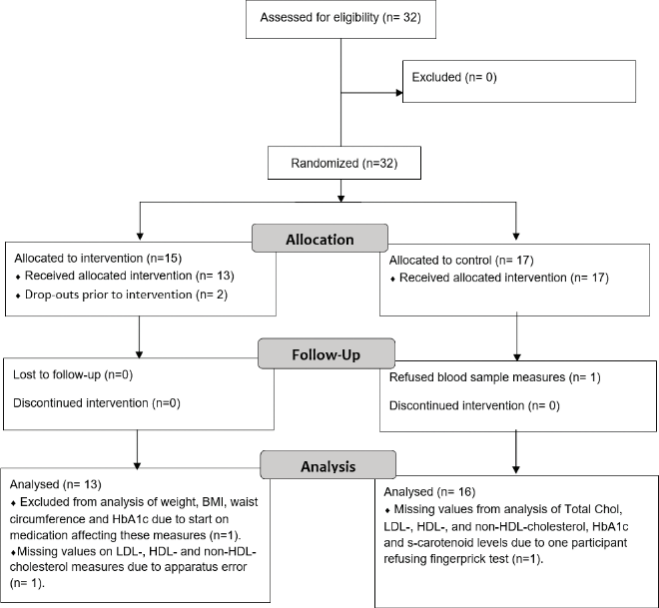
Flow diagram of enrolment of study participants.

Included adults with ID were block randomised into the intervention or control group, based on the municipal care home they lived in and the age range in different houses. The block randomisation method was selected because the intervention targeted individuals and their staff in each municipal care home and included the availability of fruits and vegetables in these units during the intervention. Randomisation was conducted by a municipality worker who was not involved in this study. The worker selected a neutral envelope from a basket containing the names of the municipal care homes. Before selecting each envelope, a decision was made on whether the municipal care home should be assigned to the intervention or control group. Five municipal care homes and 15 participants were assigned to the intervention group, and six municipal care homes and 17 participants to the control group (Fig. 1).

### Ethical standards disclosure

This study was conducted in accordance with the guidelines laid down in the Declaration of Helsinki of 1975, as revised in 2008, and all procedures involving human subjects were approved by REK Southeast (2019/362) and the Data Protection Officer at the University of Oslo. This study was registered in clinical trials with the identification number NCT04436692.

### Framework

The capability, opportunity and motivation-behaviour (COM-B) system is a comprehensive causal behavioural analysis system aimed at understanding the nature of behaviour and behaviour change to overcome possible inherent limitations of such aspects (22). Ruud et al. adapted this framework to investigate nutrition-related behaviour in persons with ID and their staff (22, 23), and this adapted model has been used to design this intervention.

### Content of the intervention

The intervention consisted of

1. Monthly practical dietary courses for people with ID and their staff.
2. Monthly separate nutrition courses for the staff.
3. *Nudging*, making fruits and vegetables available in the municipal care homes and providing different pedagogical gifts to the participants during the intervention.

1. Six dietary courses over 7 months were offered to the participants and their staff and took place once a month. Each session lasted 2 h. These courses were based on the dietary advice of the Health Directorate (24) and were converted into pictures and brand symbols for healthy food. The themes of the courses were divided into the following topics: 1) In between meals, 2) Breakfast and Supper, 3) Lunch, 4) Dinner, 5) ‘Cozy food’ and 6) a summary course to set future goals. Each course included practical cooking and tasting of simple and healthy food for each meal, and homework tasks were assigned. The tasting menu during the courses and practical tasks included the following: 1) Tasting cut-up fruits and vegetables; smoothies with fruits, berries and vegetables; and salad. 2) Tasting wholemeal bread, wholemeal crispbread and oatmeal porridge with healthy toppings and slices of fruit and vegetables. 3) Tasting a healthy lunch bag with oatmeal wraps and healthy toppings, including fruit, vegetables and water. 4) Practical tasks using the plate model to divide a small dinner plate into three parts and taste items, such as lean meat, fish, legumes, wholemeal pasta, rice, potatoes and vegetables. 5) Tasting popcorn, sugar-free soft drinks, cut-up fruit and berries, ice cream made of juice without sugar and muffins made of oatmeal, apples and blueberries. 6) Future goals were set by the participants on which of the healthy eating habits they had acquired they would concentrate on.
  At the end of each dietary course, the participants used specific BCTs (25) and set individual goals with their staff, about which food items they would eat more of, how much, how often and when. The task of the staff was to help the participants to write down their goals, to support, motivate and adapt the individual goals into routines and care plans in the municipal care homes. In addition, the participants were asked to complete a web-based e-learning nutritional course with their staff during the same period (26).
2. Once a month for 7 months, the staff attended a 2 h nutrition course. These courses took place before each dietary course with the participants. They involved teaching nutrition knowledge linked to the participant courses, providing preparative information and guiding the staff in their daily work with the participants. These courses were conducted digitally due to the COVID-19 pandemic.
3. We used *nudging* as structural tool for change in the municipal care homes. This involved posters with fruits and vegetables hanging on the walls, big salad bowls with cutlery and weekly servings of baskets of fruit and vegetables to the intervention group. Individual nudges consisted of pedagogical gifts given to the participants during the dietary courses, which were water bottles, empty lunch boxes, smoothie machines and small dinner plates (21 cm) divided into three parts with painted motifs of recommended eating, small glasses and folders with pictures, symbols and simple recipes.

### Data collection and assessment

#### Anthropometric measures and blood pressure

Weight was measured at each time point whilst wearing light clothing and recorded to the nearest 0.1 kg. A wall-mounted stadiometer was used to measure height upright and recorded to the nearest 0.1 cm. BMI was calculated using the standard formula, and standarised definitions were used to define BMI categories (27). WC was measured using a non-elastic measuring tape placed between the participants’ upper hip crest and lower rib curvatures. Gender-based standard categories were used to define categories of disease risk (27). Blood pressure was measured three times at each time point using an upper arm automatic blood pressure device and an oscillometric measurement technique (Microlife BP A100 Plus, Microlife, Widnau, Switzerland). The procedure and reference areas followed the research recommendations for BP measurements (28).

#### Assessment of dietary intake

The participants answered a short food frequency questionnaire (FFQ) with their staff about the frequency of their weekly intake of fruits, berries, vegetables, juice, soft drinks with sugar, snacks, cakes and candies. The FFQ is a simplified tool which only included these eight food groups, based on a validated form of Lillegaard et al. (29) used to assess food items amongst Norwegian children between 9 and 13 years old and previously used in a dietary intake study in adults with genetic syndromes associated with ID (10). The intake categories were less than once a week, one to three times a week, four to six times a week, daily and several times a day.

#### Carotenoid level in dried blood samples

Analyses of serum-carotenoids (s-carotenoids) (α-carotene, β-carotene, lycopene, lutein, zeaxanthin and β-cryptoxanthin), which are organic orange and red pigments from plants, were used as biomarkers in human blood for the consumption of fruits, berries and vegetables. The assessment was performed using dried finger prick blood drops placed on carotenoid sample cards for this purpose. The dried blood samples were analysed by a laboratory specialising in such analysis (Vitas Analytical Services, Oslo, Norway).

#### Lipid profile and long-time blood sugar

Non-fasting blood samples were collected from finger-prick blood drops, which were directly placed in an onsite apparatus (Afinion 2 analyser AS 100, Abbott Rapid Diagnostics AS, Norway). A non-fasting sampling of lipid parameters is recommended for general risk screening because it has the same prognostic value as fasting samples (30). The onsite apparatus measured total cholesterol, high-density lipoprotein (HDL), low-density lipoprotein (LDL), non-HDL and long-term blood sugar, glycated haemoglobin (HbA1c). International reference areas for the cholesterol variables and long-term blood sugar were used (30–32).

### Statistical analysis

Based on results from studies in other populations which had weight reduction as the primary goal of diet-oriented interventions, a weight reduction of 5 kg was expected, with a standard deviation of 4 kg (33, 34). The sample size selection was based on measures with an effect size of 1.25, an alpha level of 0.05 and a minimum desired power of (1-beta) = 0.80. Based on this, we calculated that we needed 24 participants. Dropout is typical in intervention studies; therefore, we aimed to recruit 32 participants (Fig. 1).

Standard descriptive statistics were calculated for all baseline variables (Table 1). When testing the difference between the two groups after 7 months, normality was assessed and violated for all variables except BMI. The Wilcoxon rank-sum test was used for all variables, except for BMI, where a two-sample t-test was applied (Table 2). Likewise, in the pre- and post-testing of the difference between baseline and after 7 months (Table 2), and after 14 months in the intervention group (Table 3), normality was assessed but violated for all variables, except for BMI. Therefore, the Wilcoxon signed-rank test was used for all variables except for BMI, for which a paired t-test was used.

**Table 1:** Background and baseline characteristics of the intervention- and control group

Variables	Intervention group (*n* = 13)		Control group (*n* = 17)	
	Median (%)	(Q1 – Q3)	Median	(Q1 – Q3)
Age (years)	38	(28 – 47)	43	(28 – 55)
Females (%)	11 (85)		13 (77)	
Mild grade of ID (%)	11 (85)		13 (77)	
Moderate grade (%)	2 (15)		4 (23)	
Co-located housing (%)	12 (92)		16 (94)	
Separate apartment (%)	1 (8)		1 (6)	
Self-owned apartment (%)	8 (62)		6 (35)	
Community apartment (%)	5 (38)		11 (65)	
Hours help from supporting staff	17	(13 – 20)	10	(5 – 19)
Height (cm)	156	(151 – 160)	161	(153 – 172)
Weight (kg)^†^	82.5	(74 – 107)	86	(77 – 101)
BMI (kg/m^2^)^†^	33.1	(30.1 – 38.8)	34.4	(29.0 – 41.0)
WC. (cm)^†^	104	(98 – 119)	105	(99 – 115)
Systole (mm Hg)	128	(118 – 135)	120	(110 – 130)
Diastole (mm Hg)	77	(75 – 84)	72	(67 – 79)
HbA1c (mmol/mol)^‡§^	33.5	(31.0 – 39.5)	36.5	(32.0 – 39.0)
Total chol (mmol/L)^‡¶^	5.1	(4.2 – 5.4)	5.1	(4.3 – 5.3)
LDL (mmol/L)^‡§¶^	2.8	(2.3 – 3.1)	2.5	(2.4 – 3.2)
HDL (mmol/L)^‡§¶^	1.3	(1.1 – 1.5)	1.2	(1.1 – 1.3)
Non-HDL (mmol/L)^‡§¶^	3.6	(2.8 – 4.2)	3.9	(3.2 – 4.3)
S-Carotenoids (mmol/L)^||¶^	1.2	(0.9 – 1.6)	1.3	(0.6 – 1.9)

Q1–Q3: 25^th^ and 75^th^ percentiles.

* Wilcoxon rank-sum test or Chi-square test.

† One missing value. Intervention group (*n* = 12).

‡ Samples were measured from blood drops from finger pricks.

§ Two missing values. Intervention group (*n* = 12). Control group (*n* = 16).

|| Samples were measured from dried blood drops from finger pricks on filter cards.

¶ One missing value control group (*n* = 16).

**Table 2:** Results after seven months intervention

Variables	Changes in intervention group from baseline to seven months (*n* = 13)		Changes in control group from baseline to seven months (*n* = 17)		Between the groups after seven months
	Median^†^	(95 % CI)	Median^†^	(95 % CI)	*p-* Value^‡^
Weight (kg)^¶^	-1.25	(-2.00: 0.95)	1.00	(-1.15: 3.00)	0.08
BMI (kg/m^2^)^¶^	-0.35	(-1.17: 0.36)	0.40^††^	(-0.60: 1.50)	0.07^§^
WC (cm)^¶^	-3.75**	(-7.68: -0.11)	0.00	(-3.99: 1.00)	0.03
Systole (mm Hg)	-3.00	(-7.61: 2.82)	1.00	(1.99: 5.98)	0.08
Diastole (mm Hg)	-5.00	(10.61: 2.61)	0.00	(-6.99: 5.00)	0.33
HbAlc (mmol/mol)^¶ †† ‡‡^	-0.50	(-1.89: 0.00)	1.00**	(-1.00:1.00)	0.005
Total Chol (mmol/ L)^¶ †† ‡‡ ||||^	0.21*	(-0.03: 0.62)	0.09	(-0.39: 0.76)	0.80
LDL (mmol/ L)^¶ †† ‡‡ §§ ||||^	0.07	(-0.26: 0.23)	0.36	(-0.27: 0.58)	0.68
HDL (mmol/ L)^¶ †† ‡‡ §§ ||||^	0.09	(-0.07: 0.10)	0.03	(-0.08: 0.11)	0.26
Non-HDL (mmol/L)^¶ †† ‡‡ §§ ||||^	0.25	(-0.16: 0.54)	0.16	(-0.40: 0.62)	0.63
S-Carotenoids (mmol/ L)^‡‡¶¶^	0.26**	(-0.12: 0.52)	0.12	(-0.05: 0.25)	0.11

† Wilcoxon signed-rank test.

‡ Wilcoxon rank-sum test.

§ Two-sample t-test.

|| Mean difference.

¶ One missing value in the intervention group (*n* = 12) due to start-up on medication influencing such a measure.

†† Blood samples were collected from blood drops from finger prick.

‡‡ One missing value in the control group (*n* = 16) due to refuse of taking finger prick stick.

§§ One missing value in the intervention group (*n* = 12) due to missing result from the onsite apparatus.

|||| One missing value in the control group because of start up on cholesterol lowering medication.

¶¶ Blood samples were collected from dried blood drops on filter cards from finger prick.

* *P* < 0.05,

** *P* < 0.01.

**Table 3:** Results between baseline and 14 months in the intervention group

Variables	Changes between baseline and 14 months in the intervention group (*n* = 13)		
	Median^†^	(95 % CI)	*p*- Value^‡^
Weight (kg)^¶^	-1.25	(-5.75: 0.97)	0.17
BMI (kg/m^2^)^¶^	-0.99^||^	(-2.18: 0.20)	0.09^§^
WC (cm)^¶^	-7.50	(-13.57: -3.16)	0.002
Systole (mm Hg)	-3.00	(-7.61: 2.82)	0.31
Diastole (mm Hg)	-2.00	(-4.00: 4.21)	0.59
HbAlc(mmol/mol)^¶††^	-1.00	(-2.00: 0.00)	0.15
Total Chol (mmol/L)^¶††^	0.12	(-0.15: 0.57)	0.80
LDL (mmol/L)^¶††‡‡^	-0.30	(-0.58: 0.50)	0.43
HDL (mmol/L)^¶††‡‡^	0.24	(-0.01: 0.39)	0.054
Non-HDL (mmol/L)^¶††‡‡^	-0.09	(-0.31: 0.36)	0.58
S-Carotenoids (mmol/L)^§§^	0.95	(0.15:1.11)	0.001

† Wilcoxon signed-rank test.

‡ Wilcoxon rank-sum test.

§ Two-sample t-test.

|| Mean difference.

¶ One missing value in the intervention group (*n* = 12) due to start-up on medication influencing such a measure.

†† Blood samples were collected from blood drops from finger prick.

‡‡ One missing value in the intervention group (*n* = 12) due to missing result from the onsite apparatus.

§§ Blood samples were collected from dried blood drops on filter cards from finger prick.

* *P* < 0.05,

** *P* < 0.01.

To analyse the FFQ data, answer categories of servings less than once a week, one to three times a week, four to six times a week, daily and several times a day were re-coded as numbers (0, 2, 5, 7 and 9, respectively). The Wilcoxon rank-sum test was used to determine whether there were differences between the groups after 7 months. The Wilcoxon signed-rank test was used to evaluate changes in food frequency intake from the baseline to 7 months in both groups and from the baseline to 14 months in the intervention group. Pearson’s correlation analysis was used to determine the correlation between percentage weight reduction and WC, s-carotenoid levels, cholesterol levels, HbA1c and systolic and diastolic BP after 14 months. All statistical analyses were performed using Stata 16.0 (Stata Corp LLC, Texas, USA), and *P*-values < 0.05 were regarded as statistically significant.

## Results

The background and baseline characteristics of the study population are presented in Table 1. Thirty-two participants consented and were included, with a median age of 41 years, ranging from 22 to 61 years. Two participants dropped out of the study during the COVID-19-induced delay period, but 30 participants completed the study (Fig. 1). Weight, BMI, WC and HbA1c measurements of one participant were excluded because the person concerned started Semaglutid medication during the intervention. In addition, HDL, LDL and non-HDL values are missing from one participant due to a lack of results from the onsite apparatus. Another participant refused to undergo fingerpick tests. Consequently, lipid profile, HbA1c and s-carotenoid level measurements from this person are missing throughout the study (Fig. 1).

The median number of hours of assistance per week was 15 for the entire group. Most of the participants had a mild degree of ID, and two-thirds had ID without a known cause, whereas 11 participants were diagnosed with Down syndrome. There were no significant differences between the variables measured in the two groups at baseline, neither between participants with nor without Down syndrome. Twenty-seven percent of the participants were classified as overweight with a BMI <27-29.9>, 28% as obese grade I, with a BMI <30-34.9>, 21% as obese grade II, with a BMI <35-39.9> and 24% as obese grade III, with a BMI ≤ 40. Median systolic and diastolic BP, HbA1c, total-, LDL- and HDL cholesterol levels for both the intervention and the control groups were within the normal reference area, as indicated in Table 1.

### Results after 7 months of intervention

**Fig. 2:**
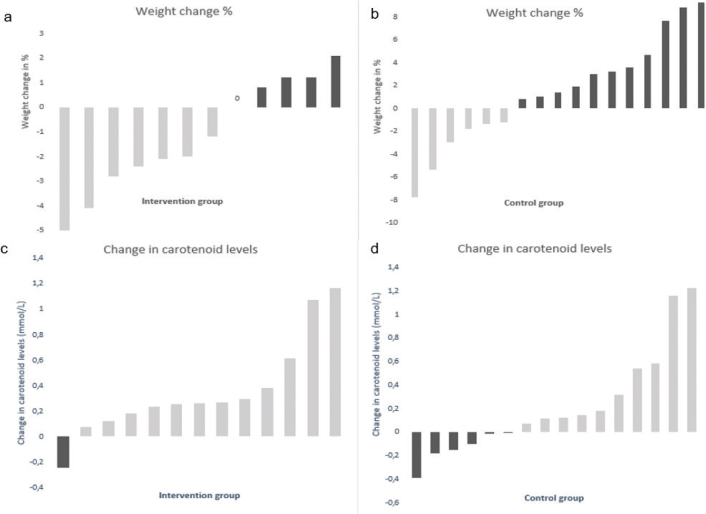
(a–d) Change in weight% and carotenoid levels after 7 months of intervention. Each bar represents participants listed according to degree of % of change and change of s-carotenoid levels. Zero represents no change.

When the primary outcome weight was analysed, more participants in the intervention group had reduced their weight (58%) compared to the control group (35%) from baseline to 7 months (Figs 2a, b). However, there was no significant difference between the intervention and control groups (*P* = 0.08, Table 2). The secondary outcome WC, however, showed a significant difference between the intervention and control groups after 7 months (*P* = 0.03, Table 2), and all participants in the intervention group reduced or maintained their WC from baseline to 7 months (*P* = 0.002, Table 2). In contrast, WC increased in over 40% of the participants in the control group (*P* = 0.51, Table 2).

Analysis of the HbA1c showed that 83% of the intervention group reduced or maintained their HbA1c levels, whereas 70% of the participants in the control group increased their HbA1c levels from baseline to 7 months. Consequently, there was a significant difference in HbA1c between the groups after 7 months of intervention (*P* = 0.003, Table 2).

Furthermore, total cholesterol was analysed and showed a significant increase in the intervention group after 7 months of intervention (*P* = 0.046, Table 2) due to a non-significant increase in median HDL cholesterol (+7.0%), LDL cholesterol (+2.5%) and non-HDL cholesterol (+7.0%). Most of the participants in the intervention group increased their s-carotenoid levels from baseline to 7 months (*P* = 0.007, Fig. 2c) in contrast to the control group, in which 35% of the participants decreased their s-carotenoid levels in the same period (*P* = 0.13, Fig. 2d).

**Fig. 3:**
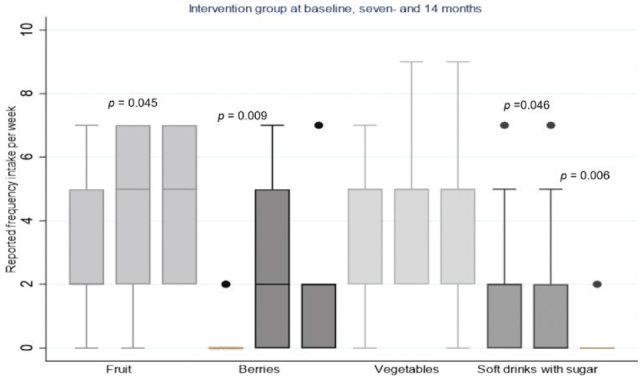
Reported servings of fruit, berries vegetables and soft drinks with sugar intake per week in the intervention group at baseline, after seven- and 14 months with their median, 25th- and 75th percentiles, minimum and maximum values and outliers. The intake frequencies are reported as median intake 0 ≤ 1 a week, 2 = 1–3 times a week, 5 = 4–6 times a week, 7 = daily and 9 = several servings a day.

Furthermore, there was a significant difference between the groups in reported FFQ intake of berries and fruit in favour of the intervention group after 7 months of intervention (median difference of berries frequency intake = 2; 95% confidence interval [CI] = 0; 3 vs. 0; 95% CI = 0; 0, *P* = 0.004, median of fruit frequency intake = 5; 95% CI = 2; 7 vs. 2; 95% CI = 2; 2, *P* = 0.03). Thirty-one percent of the intervention group reported changes in berry intake from 1–3 times or less per week to 4–6 times a week or daily in this period (median frequency intake of berries 0; 95% CI = 0; 1.2 vs. 2; 95% CI 0; 5, *P* = 0.009, Fig. 3). Furthermore, a significant reduction in the frequency of soft drinks with sugar intake was reported in the intervention group from the baseline to 7 months (median frequency intake 2; 95% CI = 0; 3.8 vs. 0; 95% CI = 0; 2, *P* = 0.046, Fig. 3).

The control group reported no significant changes in their consumption of drinks or food groups, from baseline to 7 months and after 7 months of intervention. No significant difference was found in the reported frequency intake of juice, soft drinks with sugar, snacks, cake or sweets between the intervention and the control group.

### Results after 14 months

Although two-thirds of the participants in the intervention group decreased or maintained their weight from the baseline to 14 months, there was no significant decrease in weight in the intervention group from the baseline to 14 months (*P* = 0.17, Table 3). There was, however, a significant reduction in WC from the baseline to 14 months in this group (*P* = 0.002, Table 3). The intervention group showed an increase in their HDL cholesterol levels throughout the study, with a non-significant increase after 14 months (*P* = 0.054, Table 3). Median HDL cholesterol increased (+18.5%), median LDL cholesterol decreased (−10.7%) and non-HDL cholesterol decreased (−2.5%) from the baseline to 14 months in the intervention group.

In the intervention group, a significant increase in fruit frequency intake from the baseline to 14 months was observed (median frequency fruit intake = 2; 95% CI = 0.8; 6.2 vs. 5; 95% CI = 2; 7, *P* = 0.045), together with a non-significant increase in the frequency of berry intake in the same period (median frequency berries intake = 0; 95% CI = 0; 1.2 vs. 2, 95% CI = 0; 2, *P* = 0.056). The intervention group showed a significant increase of s-carotenoid levels from the baseline to 14 months (*P* = 0.001, Table 3), and a significant reduction in the intake frequency of soft drinks with sugar intake was reported during this period (median frequency intake 2; 95% CI = 0; 3.8 vs. 0; 95% CI = 0; 0, *P* = 0.006). Regarding intake of juice, snacks, cake or sweets, no difference in intake frequency was found from the baseline to 14 months in the intervention group.

Finally, it is of interest to note that one person (8%) in the intervention group achieved a weight reduction of 5% after 7 months of intervention (Fig. 2a). Four people (33%) reduced their weight by 4.8% or more after 14 months (Table 3). Furthermore, after 14 months, a strong correlation between weight reduction of ≥4.8% was found in WC (*r* = −0.85), total cholesterol (*r* = −0.83), LDL (*r* = −0.92), HDL (*r* = 0.98) and non-HDL cholesterol (*r* = −0.95). There was a medium correlation between weight reduction and increased s-carotenoid levels (*r* = 0.40).

## Discussion

After 7 months of intervention, no significant difference in the primary outcome, weight, was observed between the intervention and the control groups (*P* = 0.08). However, a total of 58% in the intervention group reduced their weight in contrasts to 35% in the control group. At least one participant in the intervention group probably had a genetic disposition, making weight loss difficult. If this participant had been excluded from the analysis, we would have achieved significant differences in weight between the intervention group and the control group after 7-month intervention. The result observed in this study is therefore promising and needs to be followed-up in a larger cohort study. It is, however, important to underline that weight reduction is challenging to achieve in this population without combining physical activity and dietary interventions (15). Interestingly, Bergstrom et al. showed a non-significant weight reduction with a mean difference of −0.85 kg (standard deviation [SD]: 7.5) within the intervention group after 12 months of follow-up (35). Also, Lally et al. reported a weight loss of 0.02 kg (SD: 6.1) in the intervention group after 6 months of intervention (36). It is of interest to note that the secondary outcome WC showed a significant difference between the groups after 7-month intervention (*P* = 0.03) and a significant reduction in the WC in the intervention group from baseline to 7-month (−3.75 cm, *P* = 0.002), which indicate that the approach used in this study effectively reduced visceral fat and has potentials in health prevention. This study had a larger effect on reducing WC compared to the meta-analysis by Willems et al., showing an average statistical decrease in WC of = −1.69 cm (16). Increased WC is a stronger predictor than BMI of lifestyle diseases, such as cardiovascular disease, type II diabetes and some types of cancer (37).

The improvements in the secondary outcome dietary habits, with a significant increase in reported frequency intake of fruit and berries (*P* = 0.03, *P* = 0.004, respectively), supported by changes in s-carotenoid levels in the intervention group (*P* = 0.007), are in accordance with findings from another nutritional community-based health-promoting intervention study (13). Humphries et al. improved the ability to plan, prepare and serve healthy meals by staff accompanied by a significant increase in average weekly servings of vegetables (*P* = 0.03) (38). S-carotenoid levels measured in dried blood drops are objective biomarkers for the reported dietary intake frequencies of fruit, vegetables and berries. These biomarkers have been shown to correlate well with dietary intake, as measured by other assessment techniques (39, 40). Unfortunately, the measured s-carotenoids cannot be used to calculate the actual intake of fruits, berries and vegetables in the groups (41), but the frequency of intake reported in this study, especially at baseline, suggests intake below the recommended levels for such food items. These findings corroborate previous studies, which have demonstrated low intakes of fruits and vegetables in persons with ID living in municipal care homes (10, 11). The most promising result in this study was the significant increase of s-carotenoid levels estimated from the baseline to the 14 months follow-up, where no extra fruit and vegetables had been offered for the last 7 months of the study. This implies that the approach used in this study to promote dietary changes has potential and can be used to obtain long-term changes in healthy eating habits (Fig. 3).

Participants in this study reported a relatively high consumption frequency of soft drinks with sugar at the baseline, which has also been reported in other studies on persons with ID (42). High sugar intake is associated with less favourable health outcomes, such as type II diabetes, increased visceral fat and obesity in this population (42). In relation to the secondary outcome, HbA1c shows a significant difference between the groups after 7 months of intervention (*P* = 0.005). This change is closely linked to the reported reduction of soft drinks with sugar in the intervention group after 7-months (*P* = 0.046), which confirms the notion that a long-term reduction in sugary beverage consumption may improve insulin resistance, contribute to weight reduction and reduce visceral fat (43). Healthier eating patterns, such as the Mediterranean diet, have been shown to mobilise specific ectopic fat deposits and are an independent factor in reducing visceral fat (43, 44). This diet emphasises an increased intake of fruits, vegetables and less easily digestible carbohydrates, such as soft drinks with sugar. The relationship between these food items and the amount of visceral fat is consistent with our reported results.

To reduce health risks associated with obesity, weight loss between 5 and 10% is recommended (45, 46). One participant (8.3%) in the intervention group accomplished a 5% weight loss after 7 months of intervention. After 14 months, four of the intervention group participants (33.3%) had reached a 4.8% weight reduction or more. As expected, we found a correlation between weight reduction and reduction of the total LDL, non-HDL cholesterol and an increase in HDL cholesterol and s-carotenoid levels from the baseline to 14 months. Such changes in these parameters are known to reduce cardiovascular risk (31).

In this study, BCTs were used as reinforcements. It is difficult to determine which of the BCTs was most effective. However, the significant results achieved in the study confirm that this motivating way of working improves healthier eating patterns and has positive impacts on important health parameters. The role of the staff was to support and transform the goals of the participants into plans and routines in municipal care units. This is an important and positive approach to avoid challenge related to the legislation of self-determination and ethical concerns related to the use of force. These issues may be barriers to health promotion and complicate support for the work of staff (23). The nutrition courses for the staff included the possibility of reflecting on ethical dilemmas in supporting healthy behaviours for people with ID without invading individual autonomy, which is an important part of their work with people with ID (47), and other studies confirm that tailored education for residents and staff is essential in health promotion (12, 14).

The intervention took place during the COVID-19 pandemic when society was shut down, and lives of participants were restricted. Leisure activities were shut down, such as attending the gym, swimming hall or participating in football or other leisure activities. All the participants were restricted from visiting relatives and friends or spending time with their support contacts, and their workplaces were shut down. As a result, the participants lost the opportunity to walk back and forth to their work or use public transport. Consequently, many participants were less active during the 7 months of intervention. The restrictions for physical activity probably had a significant negative impact on all the outcome variables, such as weight loss, WC, HbA1c and cholesterol levels, as physical activity is a well-known influence on these measures (30–32). Fewer opportunities for leisure activities and lack of attending work may also have increased snacking. The significant increase in HbA1c and the weight increase in the control group during the intervention period support this adverse effect of inactivity and snacking due to the pandemic (32, 42). Therefore, the COVID-19 pandemic may, to some extent, have counteracted some of the positive effects of the intervention.

This study has several limitations. The small study sample increases the risk of type II errors and affected the possibility of performing subgroup analysis. Furthermore, many participants had a BMI of >35, and some had comorbidities with underlying dispositions for lifestyle diseases, which this study was not designed to address. In addition, some needed specially adapted diets, such as gluten-free, for which the diet courses were not designed.

The FFQ used for the assessment of dietary intakes only captures intake frequency of selected food groups, and there are distinct limitations to this approach to assess changes in dietary habits. Even so, for the food groups assessed, the data reported show consistency between the frequency of reported intake and the measured biomarkers, strengthening the validity of the data obtained.

One person performed all measurements of the participants; although this is a strength related to internal validity, this could potentially result in assessment bias. Finally, as discussed earlier, the possible effects of social isolation due to the COVID-19 pandemic may have significant impact on the intervention results. Therefore, a key limitation of this study is that it did not control for physical activity. Nevertheless, our results provide a foundation for a larger RCT with a similar methodology on a more homogeneous study population to provide more detailed and targeted data on this population at a time without the restrictions imposed by the COVID-19 pandemic.

## Conclusion

A significant difference in WC, but not on body weight, was observed between the intervention group and the control group after 7 months of intervention. This was accompanied by a significant difference between the groups in reported frequency of fruit and berry intake. Furthermore, there was a significant difference in HbA1c levels between the groups after 7 months of intervention. The intervention group, following a 7-month follow-up period with usual care, showed a significant reduction in their WC and consumption frequency of soft drinks with sugar, along with a significant increase in consumption frequency of fruits and s-carotenoid levels. These results indicate that this multi-component RCT can reduce visceral fat and improve relevant blood parameters and dietary habits. The effects observed in this study are promising and should be evaluated further in a larger study population of adults with mild ID.
